# Ethyl (2*E*)-2-cyano-3-(1-methyl-1*H*-pyrrol-2-yl)prop-2-enoate

**DOI:** 10.1107/S1600536811031941

**Published:** 2011-08-11

**Authors:** Abdullah M. Asiri, Abdulrahman O. Al-Youbi, Khalid A. Alamry, Hassan M. Faidallah, Seik Weng Ng, Edward R. T. Tiekink

**Affiliations:** a Chemistry Department, Faculty of Science, King Abdulaziz University, PO Box 80203, Jeddah, Saudi Arabia; b The Center of Excellence for Advanced Materials Research, King Abdulaziz University, Jeddah, PO Box 80203, Saudi Arabia; c Department of Chemistry, University of Malaya, 50603 Kuala Lumpur, Malaysia

## Abstract

The 15 non-H atoms of the title compound, C_11_H_12_N_2_O_2_, are approximately coplanar, the r.m.s. deviation being 0.145 Å. The major deviation from coplanarity is seen in a twist between the ethene (*E* configuration) and pyrrole rings [C—C—N—C torsion angle = −8.26 (18)°]. The carbonyl O and cyano N atoms are *syn* to each other. In the crystal, supra­molecular linear tapes linked by C—H⋯O and C—H⋯N inter­actions are further connected by C—H⋯π(pyrrole) inter­actions.

## Related literature

For background to the biological activity of 2(1*H*)pyridone compounds, see: Aly *et al.* (1991 ▶); Al-Saadi *et al.* (2005 ▶); Rostom *et al.* (2011 ▶).
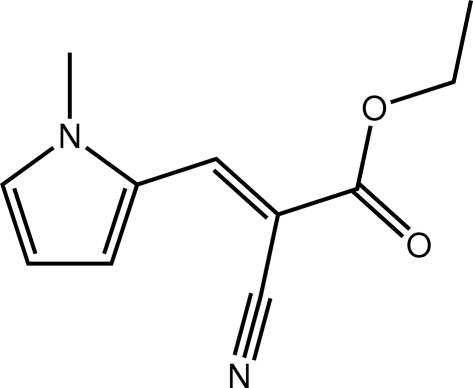


## Experimental

### 

#### Crystal data

C_11_H_12_N_2_O_2_
*M* *_r_* = 204.23
Triclinic, 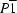

*a* = 7.6145 (3) Å
*b* = 8.4964 (6) Å
*c* = 9.7023 (6) Å
α = 64.898 (7)°
β = 89.859 (4)°
γ = 71.517 (5)°
*V* = 532.69 (5) Å^3^
*Z* = 2
Mo *K*α radiation
μ = 0.09 mm^−1^
*T* = 100 K
0.30 × 0.25 × 0.10 mm

#### Data collection

Agilent SuperNova Dual diffractometer with an Atlas detector
Absorption correction: multi-scan (*CrysAlis PRO*; Agilent, 2010 ▶) *T*_min_ = 0.955, *T*_max_ = 1.000
4049 measured reflections
2336 independent reflections
1912 reflections with *I* > 2σ(*I*)
*R*_int_ = 0.030

#### Refinement

*R*[*F* ^2^ > 2σ(*F* ^2^)] = 0.041
*wR*(*F* ^2^) = 0.106
*S* = 1.04
2336 reflections
138 parameters
H-atom parameters constrained
Δρ_max_ = 0.26 e Å^−3^
Δρ_min_ = −0.21 e Å^−3^

### 

Data collection: *CrysAlis PRO* (Agilent, 2010 ▶); cell refinement: *CrysAlis PRO*; data reduction: *CrysAlis PRO*; program(s) used to solve structure: *SHELXS97* (Sheldrick, 2008 ▶); program(s) used to refine structure: *SHELXL97* (Sheldrick, 2008 ▶); molecular graphics: *ORTEP-3* (Farrugia, 1997 ▶) and *DIAMOND* (Brandenburg, 2006 ▶); software used to prepare material for publication: *publCIF* (Westrip, 2010 ▶).

## Supplementary Material

Crystal structure: contains datablock(s) global, I. DOI: 10.1107/S1600536811031941/hb6354sup1.cif

Structure factors: contains datablock(s) I. DOI: 10.1107/S1600536811031941/hb6354Isup2.hkl

Additional supplementary materials:  crystallographic information; 3D view; checkCIF report

## Figures and Tables

**Table 1:** Hydrogen-bond geometry (Å, °) *Cg*1 is the centroid of the N2,C7—C10 ring.

*D*—H⋯*A*	*D*—H	H⋯*A*	*D*⋯*A*	*D*—H⋯*A*
C11—H11a⋯O2^i^	0.98	2.31	3.241 (2)	158
C9—H9⋯N1^ii^	0.95	2.62	3.557 (2)	171
C11—H11b⋯*Cg*1^iii^	0.98	2.69	3.5332 (17)	144

Symmetry codes: (i) 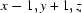; (ii) 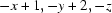; (iii) 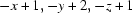.

**Fig. 1.**
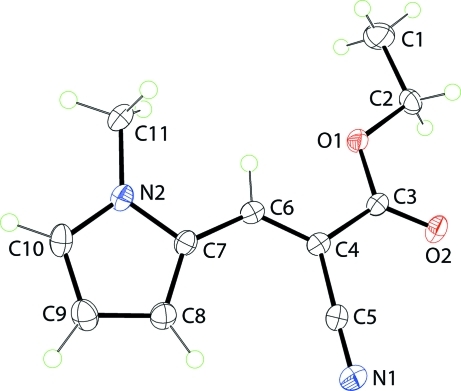
The molecular structure of (I) showing displacement ellipsoids at the 50% probability level.

**Fig. 2.**
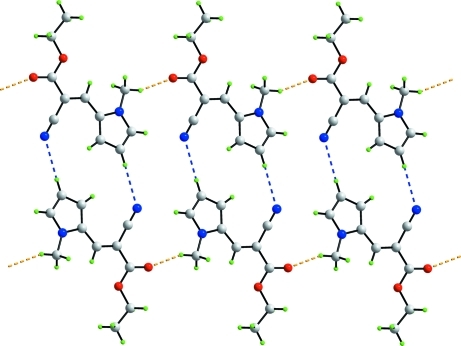
Supramolecular tape in (I) mediated by C—H···O and C—H···N interactions shown as orange and blue dashed lines, respectively.

**Fig. 3.**
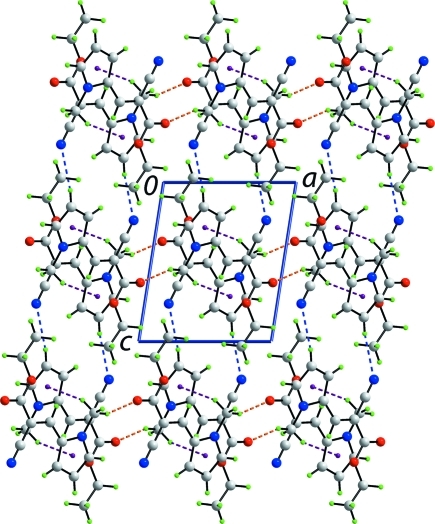
A view in projection down the b axis of the unit-cell contents of (I). The C—H···O, C—H···N and C—H···π interactions shown as orange, blue and purple dashed lines, respectively.

## supplementary crystallographic information

### Comment

The title compound (I) was studied in connection with the known biological activity of 2(1*H*)pyridone compounds (Aly *et al.*, 1991; Al-Saadi *et al.*, 2005; Rostom *et al.*, 2011), and was prepared from the condensation of the *N*-methylpyrrole-2-carboxaldehyde with ethyl cyanoacetate during an attempt to prepare a 2(1*H*)pyridone derivative.

The molecular structure of (I), Fig. 1, is, to a first approximation, planar with the r.m.s. deviation for all 15 non-H atoms being 0.145 Å. The major deviations from the least-squares plane are 0.214 (2) and -0.337 (2) Å for the C9 and C11 atoms, respectively, reflecting a small twist between the ethene and pyrrole rings [the C11—N2—C7—C6 torsion angle = -8.26 (18) °]. The conformation about the ethene [C4═C7 = 1.3594 (18) Å] bond is *E*. The carbonyl-O and cyano-N atoms are *syn* to each other.

In the crystal packing, molecules are linked into chains *via* C—H···O interactions involving a *N*-bound methyl-H and carbonyl-O, Table 1. Chains are linked into a linear tape *via* C—H···N interactions involving a pyrrole-H and cyano-N, Fig. 2. The tapes are consolidated into the three-dimensional architecture by C—H···π interactions, Fig. 3, involving another *N*-bound methyl-H as the donor to the pyrrole ring.

### Experimental

A mixture of the *N*-methylpyrrole-2-carboxaldehyde (1.0 g,1 0 mmol), 2-methylcyclohexanone (1.12 g, 10 mmol), ethyl cyanoacetate (1.1 g, 10 mmol) and ammonium acetate (6.2 g, 80 mmol) in absolute ethanol (50 ml) was refluxed for 6 h. The reaction mixture was allowed to cool, the formed precipitate was filtered, washed with water, dried and recrystallized from ethanol to form yellow blocks. *M*.pt. 420–421 K.

### Refinement

Carbon-bound H-atoms were placed in calculated positions [C—H 0.95 to 0.99 Å, *U*_iso_(H) 1.2 to 1.5*U*_eq_(C)] and were included in the refinement in the riding model approximation.

### Crystal data

**Table:**

C_11_H_12_N_2_O_2_	*Z* = 2
*M**_r_* = 204.23	*F*(000) = 216
Triclinic, *P*1	*D*_x_ = 1.273 Mg m^−^^3^
Hall symbol: -P 1	Mo *K*α radiation, λ = 0.71073 Å
*a* = 7.6145 (3) Å	Cell parameters from 2042 reflections
*b* = 8.4964 (6) Å	θ = 2.3–29.2°
*c* = 9.7023 (6) Å	µ = 0.09 mm^−^^1^
α = 64.898 (7)°	*T* = 100 K
β = 89.859 (4)°	Block, yellow
γ = 71.517 (5)°	0.30 × 0.25 × 0.10 mm
*V* = 532.69 (5) Å^3^	

### Data collection

**Table:**

Agilent SuperNova Dual diffractometer with an Atlas detector	2336 independent reflections
Radiation source: SuperNova (Mo) X-ray Source	1912 reflections with *I* > 2σ(*I*)
mirror	*R*_int_ = 0.030
Detector resolution: 10.4041 pixels mm^-1^	θ_max_ = 27.5°, θ_min_ = 2.4°
ω scans	*h* = −8→9
Absorption correction: multi-scan (*CrysAlis PRO*; Agilent, 2010)	*k* = −8→11
*T*_min_ = 0.955, *T*_max_ = 1.000	*l* = −12→11
4049 measured reflections	

### Refinement

**Table:**

Refinement on *F*^2^	Primary atom site location: structure-invariant direct methods
Least-squares matrix: full	Secondary atom site location: difference Fourier map
*R*[*F*^2^ > 2σ(*F*^2^)] = 0.041	Hydrogen site location: inferred from neighbouring sites
*wR*(*F*^2^) = 0.106	H-atom parameters constrained
*S* = 1.04	*w* = 1/[σ^2^(*F*_o_^2^) + (0.043*P*)^2^ + 0.1378*P*] where *P* = (*F*_o_^2^ + 2*F*_c_^2^)/3
2336 reflections	(Δ/σ)_max_ < 0.001
138 parameters	Δρ_max_ = 0.26 e Å^−^^3^
0 restraints	Δρ_min_ = −0.21 e Å^−^^3^

### Special details

**Table:**

Geometry. All e.s.d.'s (except the e.s.d. in the dihedral angle between two l.s. planes) are estimated using the full covariance matrix. The cell e.s.d.'s are taken into account individually in the estimation of e.s.d.'s in distances, angles and torsion angles; correlations between e.s.d.'s in cell parameters are only used when they are defined by crystal symmetry. An approximate (isotropic) treatment of cell e.s.d.'s is used for estimating e.s.d.'s involving l.s. planes.
Refinement. Refinement of *F*^2^ against ALL reflections. The weighted *R*-factor *wR* and goodness of fit *S* are based on *F*^2^, conventional *R*-factors *R* are based on *F*, with *F* set to zero for negative *F*^2^. The threshold expression of *F*^2^ > σ(*F*^2^) is used only for calculating *R*-factors(gt) *etc*. and is not relevant to the choice of reflections for refinement. *R*-factors based on *F*^2^ are statistically about twice as large as those based on *F*, and *R*- factors based on ALL data will be even larger.

### Fractional atomic coordinates and isotropic or equivalent isotropic displacement parameters (Å^2^)

**Table:**

	*x*	*y*	*z*	*U*_iso_*/*U*_eq_	
O1	0.76560 (13)	0.39008 (13)	0.75762 (10)	0.0210 (2)	
O2	0.94151 (13)	0.24631 (13)	0.62962 (11)	0.0240 (2)	
N2	0.31730 (15)	0.98384 (16)	0.40412 (13)	0.0195 (3)	
C1	0.7806 (2)	0.2663 (2)	1.02981 (18)	0.0367 (4)	
H1A	0.8394	0.1579	1.1287	0.055*	
H1B	0.6452	0.2928	1.0165	0.055*	
H1C	0.8049	0.3737	1.0280	0.055*	
C2	0.8606 (2)	0.22752 (19)	0.90199 (16)	0.0239 (3)	
H2A	0.9970	0.2036	0.9128	0.029*	
H2B	0.8402	0.1170	0.9045	0.029*	
C3	0.82056 (17)	0.37907 (18)	0.63059 (15)	0.0178 (3)	
C4	0.71681 (17)	0.54814 (18)	0.48907 (15)	0.0177 (3)	
C5	0.77316 (18)	0.54540 (18)	0.34926 (16)	0.0198 (3)	
C6	0.57429 (17)	0.68970 (18)	0.49404 (15)	0.0176 (3)	
H6	0.5455	0.6706	0.5940	0.021*	
C7	0.46245 (17)	0.86137 (18)	0.37221 (15)	0.0185 (3)	
C8	0.47051 (19)	0.95100 (19)	0.21554 (16)	0.0225 (3)	
H8	0.5559	0.9015	0.1603	0.027*	
C9	0.3314 (2)	1.1258 (2)	0.15416 (17)	0.0268 (3)	
H9	0.3051	1.2171	0.0499	0.032*	
C10	0.23870 (19)	1.14206 (19)	0.27246 (16)	0.0241 (3)	
H10	0.1364	1.2471	0.2631	0.029*	
C11	0.25065 (19)	0.9441 (2)	0.55193 (16)	0.0230 (3)	
H11A	0.1556	1.0568	0.5462	0.034*	
H11B	0.3559	0.9013	0.6323	0.034*	
H11C	0.1956	0.8473	0.5767	0.034*	
N1	0.81855 (17)	0.53952 (17)	0.23809 (14)	0.0273 (3)	

### Atomic displacement parameters (Å^2^)

**Table:**

	*U*^11^	*U*^22^	*U*^33^	*U*^12^	*U*^13^	*U*^23^
O1	0.0231 (5)	0.0179 (5)	0.0169 (5)	−0.0016 (4)	0.0022 (4)	−0.0073 (4)
O2	0.0218 (5)	0.0215 (5)	0.0278 (6)	−0.0014 (4)	0.0028 (4)	−0.0145 (4)
N2	0.0176 (5)	0.0198 (6)	0.0224 (6)	−0.0041 (5)	0.0011 (4)	−0.0122 (5)
C1	0.0394 (9)	0.0389 (10)	0.0209 (8)	−0.0062 (8)	0.0046 (7)	−0.0089 (7)
C2	0.0269 (7)	0.0183 (7)	0.0187 (7)	−0.0043 (6)	−0.0005 (6)	−0.0038 (6)
C3	0.0163 (6)	0.0193 (7)	0.0206 (7)	−0.0061 (5)	0.0036 (5)	−0.0116 (6)
C4	0.0167 (6)	0.0202 (7)	0.0194 (7)	−0.0076 (5)	0.0035 (5)	−0.0108 (6)
C5	0.0183 (6)	0.0179 (7)	0.0225 (7)	−0.0044 (5)	0.0019 (5)	−0.0098 (6)
C6	0.0169 (6)	0.0211 (7)	0.0190 (7)	−0.0084 (5)	0.0037 (5)	−0.0115 (5)
C7	0.0174 (6)	0.0190 (7)	0.0208 (7)	−0.0054 (5)	0.0019 (5)	−0.0111 (6)
C8	0.0254 (7)	0.0231 (7)	0.0204 (7)	−0.0069 (6)	0.0011 (5)	−0.0120 (6)
C9	0.0305 (8)	0.0234 (8)	0.0213 (7)	−0.0055 (6)	−0.0048 (6)	−0.0082 (6)
C10	0.0211 (7)	0.0194 (7)	0.0282 (8)	−0.0012 (6)	−0.0045 (6)	−0.0115 (6)
C11	0.0199 (7)	0.0251 (8)	0.0283 (8)	−0.0059 (6)	0.0061 (6)	−0.0172 (6)
N1	0.0325 (7)	0.0267 (7)	0.0234 (7)	−0.0074 (6)	0.0076 (5)	−0.0139 (5)

### Geometric parameters (Å, °)

**Table:**

O1—C3	1.3316 (16)	C4—C5	1.4290 (19)
O1—C2	1.4570 (16)	C5—N1	1.1482 (17)
O2—C3	1.2128 (15)	C6—C7	1.4158 (18)
N2—C10	1.3542 (18)	C6—H6	0.9500
N2—C7	1.3938 (17)	C7—C8	1.3933 (19)
N2—C11	1.4568 (18)	C8—C9	1.392 (2)
C1—C2	1.494 (2)	C8—H8	0.9500
C1—H1A	0.9800	C9—C10	1.380 (2)
C1—H1B	0.9800	C9—H9	0.9500
C1—H1C	0.9800	C10—H10	0.9500
C2—H2A	0.9900	C11—H11A	0.9800
C2—H2B	0.9900	C11—H11B	0.9800
C3—C4	1.4783 (18)	C11—H11C	0.9800
C4—C6	1.3594 (18)		
			
C3—O1—C2	115.44 (10)	N1—C5—C4	178.60 (15)
C10—N2—C7	108.92 (11)	C4—C6—C7	129.52 (13)
C10—N2—C11	125.09 (11)	C4—C6—H6	115.2
C7—N2—C11	125.83 (11)	C7—C6—H6	115.2
C2—C1—H1A	109.5	C8—C7—N2	106.74 (11)
C2—C1—H1B	109.5	C8—C7—C6	133.53 (12)
H1A—C1—H1B	109.5	N2—C7—C6	119.56 (12)
C2—C1—H1C	109.5	C9—C8—C7	107.97 (13)
H1A—C1—H1C	109.5	C9—C8—H8	126.0
H1B—C1—H1C	109.5	C7—C8—H8	126.0
O1—C2—C1	107.45 (11)	C10—C9—C8	107.48 (13)
O1—C2—H2A	110.2	C10—C9—H9	126.3
C1—C2—H2A	110.2	C8—C9—H9	126.3
O1—C2—H2B	110.2	N2—C10—C9	108.89 (12)
C1—C2—H2B	110.2	N2—C10—H10	125.6
H2A—C2—H2B	108.5	C9—C10—H10	125.6
O2—C3—O1	124.42 (12)	N2—C11—H11A	109.5
O2—C3—C4	123.32 (12)	N2—C11—H11B	109.5
O1—C3—C4	112.26 (11)	H11A—C11—H11B	109.5
C6—C4—C5	123.65 (12)	N2—C11—H11C	109.5
C6—C4—C3	121.69 (12)	H11A—C11—H11C	109.5
C5—C4—C3	114.60 (11)	H11B—C11—H11C	109.5
			
C3—O1—C2—C1	179.92 (11)	C10—N2—C7—C6	176.08 (11)
C2—O1—C3—O2	−0.45 (18)	C11—N2—C7—C6	−8.26 (18)
C2—O1—C3—C4	179.47 (10)	C4—C6—C7—C8	−8.2 (2)
O2—C3—C4—C6	175.93 (12)	C4—C6—C7—N2	177.34 (12)
O1—C3—C4—C6	−3.99 (17)	N2—C7—C8—C9	0.03 (15)
O2—C3—C4—C5	−1.38 (18)	C6—C7—C8—C9	−174.98 (14)
O1—C3—C4—C5	178.70 (10)	C7—C8—C9—C10	−0.29 (16)
C5—C4—C6—C7	−4.1 (2)	C7—N2—C10—C9	−0.43 (15)
C3—C4—C6—C7	178.85 (12)	C11—N2—C10—C9	−176.13 (12)
C10—N2—C7—C8	0.24 (14)	C8—C9—C10—N2	0.44 (16)
C11—N2—C7—C8	175.90 (11)		

### Hydrogen-bond geometry (Å, °)

**Table:**

Cg1 is the centroid of the N2,C7—C10 ring.

**Table:**

*D*—H···*A*	*D*—H	H···*A*	*D*···*A*	*D*—H···*A*
C11—H11a···O2^i^	0.98	2.31	3.241 (2)	158
C9—H9···N1^ii^	0.95	2.62	3.557 (2)	171
C11—H11b···Cg1^iii^	0.98	2.69	3.5332 (17)	144

Symmetry codes: (i) *x*−1, *y*+1, *z*; (ii) −*x*+1, −*y*+2, −*z*; (iii) −*x*+1, −*y*+2, −*z*+1.
